# The Utilization of Dental Services Among Indonesian Youth: A Nationwide Study Based on the Expanded Andersen Behavioral Model

**DOI:** 10.1016/j.identj.2026.109784

**Published:** 2026-08-08

**Authors:** Herry Novrinda, Shifa Qudsialaiqa Irsan, Atik Ramadhani, Risqa Rina Darwita, Han Dong-Hun, Iwany Amalliah Badruddin, Armasastra Bahar

**Affiliations:** a Department of Dental Public Health and Preventive Dentistry, Faculty of Dentistry, University of Indonesia, Jakarta, Indonesia; b Faculty of Dentistry, University of Indonesia, Jakarta, Indonesia; c Department of Preventive and Social Dentistry, Seoul National University School of Dentistry, Seoul, South Korea; d Dental Research Institute, Seoul National University, Seoul, South Korea

**Keywords:** Indonesian Health Survey 2023, Dental services, Youth, Anderson Behavioral Model

## Abstract

**Background:**

The purpose of this study was to examine factors associated with dental service utilization among Indonesian youth using the Expanded Andersen Behavioral Model (EABM).

**Methods:**

This cross-sectional study assessed data from 3332 people aged 15 to 24 from the 2023 Indonesian Health Survey. Using EABM, variables were classified as predisposing, enabling, need, and psychosocial domains. Dental service utilization within the previous year was the outcome variable. A series of appropriate statistical analysis, such as chi-square, binary logistic regression, multivariable logistic regression, and principal component analysis (PCA) with varimax rotation was conducted to analyse the 2023 Indonesian Health Survey data. Logistic regressions were reported as odds ratios (ORs) with 95% confidence intervals (CIs). PCA was conducted to examine clustering patterns among the included variables and to assess whether these patterns were broadly consistent with the conceptual domains.

**Results:**

Overall, only 7.6% of respondents reported using dental services within the previous year. Female (adjusted OR [AOR] [95% CI] = 1.655 [1.236-2.154]), urban residence (AOR [95% CI] = 2.274 [1.689-3.058]), and health insurance ownership (AOR [95% CI] = 1.985 [1.383-2.849]) were significantly associated with higher utilization rates. Factors that showed the strongest associations were oral pain (AOR [95% CI] = 12.630 [7.747-20.582]) and caries experience (AOR [95% CI] = 2.342 [1.467-3.741]). In contrast, psychosocial factors, including depression and psychological distress, were not significantly associated with utilization. PCA identified four components corresponding to psychosocial, need, predisposing, and enabling domains, which explained 60.24% of total variance, supporting the conceptual structure of EABM.

**Conclusion:**

Dental service utilization among Indonesian youth remains low and is primarily driven by clinical need and structural access factors rather than psychosocial conditions. While psychosocial variables were in line with a coherent latent construct, they did not translate into observable utilization behaviour.

## Introduction

Oral diseases remain a major public health concern worldwide and disproportionately affect adolescents and young adults. Dental caries, periodontal diseases, and untreated oral conditions may negatively influence quality of life, educational performance, social interaction, and overall well-being. World Health Organization has identified oral diseases as among the most prevalent noncommunicable diseases globally, affecting nearly 3.5 billion people worldwide.^1^ Untreated dental caries alone affects approximately 2.3 billion individuals globally and remains highly prevalent among adolescents and young adults.^2^

Despite advances in preventive dentistry and oral health promotion, utilization of dental health services among young populations remains suboptimal in many low- and middle-income countries, including Indonesia. Regular dental visits are essential not only for treatment of oral disease but also for prevention, early diagnosis, and maintenance of long-term oral health. However, adolescents and young adults frequently seek dental care only when symptoms become severe, reflecting a predominantly curative rather than preventive utilization pattern.^3^

In 2009, the Ministry of Health of the Republic of Indonesia categorized adolescents into two categories: those aged 12 to 16 as early adolescents and those aged 17 to 25 as late adolescents. The Indonesian Health Survey 2023 (IHS 2023) regarded the 10 to 14 age group as early adolescents and the 15 to 24 age group as late adolescents or youth (*Report of the Advisory Committee for the International Youth Year (A/36/215 annex*).

Indonesia continues to face substantial challenges in oral healthcare utilization despite implementation of the national health insurance system (Jaminan Kesehatan Nasional/JKN). A previous population-based study in Indonesia reported that most individuals had never visited a dentist within the previous year, despite high prevalence of oral diseases.^4^ Structural inequalities in access to care, unequal distribution of oral healthcare providers, limited oral health literacy, and socioeconomic barriers may contribute to underutilization,^5^ particularly among younger populations and rural communities. Understanding determinants of dental service utilization among youth is therefore important for developing evidence-based oral health policies and preventive strategies.

Healthcare utilization behaviour is commonly explained using the Andersen and Newman Behavioral Model, which conceptualizes healthcare utilization as being influenced by predisposing, enabling, and need factors.^6^ The Expanded Andersen Behavioral Model (EABM) further incorporates psychosocial dimensions, including psychological distress and mental health-related factors, to better explain healthcare-seeking behaviour.^7^^,^^8^ Previous studies applying the Andersen framework have demonstrated that utilization of dental services may be influenced by demographic characteristics, financial access, oral health needs, and psychosocial conditions.^3^^,^^9^ A systematic review based on the Andersen model further concluded that need factors and enabling resources consistently represent the strongest determinants of dental service utilization across populations.^9^ Nevertheless, findings regarding psychosocial influences remain inconsistent across settings and populations.

Most previous studies on dental service utilization have been conducted in high-income countries, whereas evidence from low- and middle-income countries remains comparatively limited.^9^ In addition, few studies have simultaneously examined psychosocial, structural, and clinical determinants of dental service utilization among youth populations using nationally representative data.^3^^,^^10^^,^^11^ Within the Indonesian context, understanding how these domains interact is particularly important because oral healthcare utilization may be strongly associated with structural access barriers and symptom-driven treatment-seeking behaviour.

This study extends applications of EABM by examining how Indonesia’s evolving healthcare system, including national health insurance coverage and rural–urban disparities, relates to dental service utilization among youth. In addition, this study evaluates whether psychosocial variables in line with behavioural constructs and whether these constructs translate into observable utilization behaviour. Therefore, this study also aimed to identify factors associated with utilization of dental health services among Indonesian youth using data from the 2023 IHS.

## Material and methods

### Study population and design

This research conducted a secondary analysis of data from the IHS 2023.^12^ IHS 2023, a cross-sectional nationwide survey, constituted a segment of the serial Indonesian nationwide Basic Health Survey conducted quinquennially. IHS 2023 employed stratification at both the bloc census and household levels within the chosen bloc to obtain a representative sample. Indonesia was divided into urban and rural regions. Initially, multiple blocks from the census were chosen using probability proportionate to size. Secondly, many families were chosen using systematic sampling with implicit stratification based on the educational attainment of the household head.

### Data collection and management

#### Study variables

Data IHS 2023 were collected through a questionnaire in the Indonesian language and by a clinical dental examination. The inclusion criteria in this study were that the respondents had to be between 15 and 24 years old and to have answered the questionnaire by themselves and undergone a clinical oral examination.

#### Dependent variable

The dependent variable or outcome of interest in this study was the utilization of oral health services, which was self-reported by respondents by answering a single question: ‘*Have you visited a dental health practitioner in the last year?*’ The response options were *yes or no*. According to the American and Irish Dental Association, question regarding the use of oral health services in the past year is commonly used to obtain this information.^13^

#### Independent variable

The independent variables or potential indicators of oral health service utilization in this study were organised according to the Andersen and Newman framework, including predisposing, enabling, and need factors.^6^^,^^14^ Predisposing factors that included gender (female or male), residential area (urban or rural), marital status (married or divorced/single). Enabling factors were health insurance ownership (have or not have), employment status (employed or unemployed). Needs factors were oral pain in the previous year (yes or no) and caries experience based on clinical examination (have caries or not). Psychological factors were depression condition (no depression or had depression), psychological distress (no psychological distress or had psychological distress).

### Statistical analysis

Data analysis was conducted utilizing IBM SPSS Statistics version 30 (IBM Corp). Descriptive statistics were employed to encapsulate respondent characteristics and patterns of dental service utilization. The chi-square test was employed to examine associations between independent variables and dental service utilization. A binary logistic regression and multivariable-adjusted logistic regression analysis were performed to estimate crude and adjusted odds ratios (ORs) and 95% confidence intervals (CIs) for factors related to the utilization of dental health services. Statistical significance was determined at *P* < .05. To assess potential multicollinearity among independent variables, variance inflation factors (VIFs) and tolerance statistics were examined. Consistent with previous recommendations, VIF values below 5 and tolerance values above 0.20 were considered indicative of the absence of problematic multicollinearity.^15^^,^^16^

According to EABM, variables were classified into predisposing, enabling, need, and psychosocial domains. Principal component analysis (PCA) with varimax rotation was conducted to assess the underlying structure and conceptual categorization of these variables. The adequacy of sampling was evaluated by the *Kaiser–Meyer–Olkin* (KMO) measure and Bartlett’s test of sphericity. Components with eigenvalues exceeding 1.0 were preserved, and factor loadings of 0.40 or greater were deemed significant for interpretation. PCA was conducted to examine clustering patterns among the included variables and to assess whether these patterns were broadly consistent with the conceptual domains of the EABM.

### Ethical review

Permission to use the IHS 2023 data was granted by the Ministry of Health of the Republic of Indonesia’s Human Research Ethics Committee. The respondents were allocated code numbers to ensure anonymization. Since only secondary data were used, this study was ethically exempted (***no. 24/Ethical Exempted/FKGUI/IX/2024***) by the Dentistry Research Ethics Committee, Faculty of Dentistry, University of Indonesia.

## Results

Of the 3332 respondents, only 252 (7.6%) had visited a dental health practitioner in the previous year, indicating a low level of dental service utilization among Indonesian youth. Most of the respondents were female (57.3%), were living in urban areas (57.8%), were divorced or single (82.6%), had health insurance (73.9%), were unemployed (76.0%), had oral pain in the previous year (53.8%), and had caries experience (79.6%), did not have depression (96.4%) and did not have psychological distress (95.7%).

### Association between independent variables and dental service utilization

Table 1 presents the distribution, associations, and crude ORs (COR) for factors associated with dental visits in the previous year. Among the predisposing factors, the female group had higher probability to visit a dental health practitioner than male (COR [95% CI] = 1.662 [1.263-2.188]; *P* < .001). Living in urban areas had higher probability to visit a dental health practitioner than living in rural ones (COR [95% CI] = 1.989 [1.496-2.644]**;** *P* < .001). Marital status was not significantly associated with dental service utilization (*P* = .597). Among enabling factors, respondents with health insurance were significantly more likely to utilize dental services than uninsured respondents (COR [95% CI] = 2.001 [1.410-2.842]; *P* < .001). In contrast, employment status was not significantly associated with dental visits (*P* = .702). Need factors demonstrated the strongest associations with dental service utilization. Respondents who experienced oral pain in the previous year were substantially more likely to visit dental services than those without oral pain (COR [95% CI] = 12.700 [7.822-20.618]; *P* < .001). Likewise, respondents with caries experience were significantly more likely to seek dental care (COR [95% CI] = 3.000 [1.903-4.728]; *P* < .001). Regarding psychosocial factors, neither depression nor psychological distress showed statistically significant associations with dental service utilization. Respondents with depression had similar utilization patterns to those without depression (COR [95% CI] = 1.000 [0.501-1.998]; *P* = 1.000), while psychological distress was also not significantly associated with dental visits (COR [95% CI] = 1.472 [0.848-2.554]; *P* = .169).

**Table 1:** Demographic, association, crude ORs between dental visits in the previous year and independent variables of youth respondents in the 2023 Indonesian Health Survey.

Variables		Dental visit in the previous year (y)		*P* value*	Crude OR (95% CI)†
	Total (%) (*n* = 3332)	Not visited	Visited		
**Predisposing factors**					
**Gender**					
Female	1909 (57.3)	1737 (91)	172 (9)	<.001*	**1.662 (1.263-2.188)**
Male (ref)	1423 (42.7)	1343 (94.4)	80 (5.6)		1
**Residential area**					
Urban	1927 (57.8)	1745 (90.6)	182 (9.4)	<.001*	**1.989 (1.496-2.644)**
Rural (ref)	1405 (42.2)	1335 (95)	70 (5.0)		1
Marital status					
Married	581 (17.4)	534 (91.9)	47 (8.1)	.597	1.093 (0.786-1.521)
Divorced/single (ref)	2751 (82.6)	2546 (92.5)	205 (7.5)		1
**Enabling factors**					
**Health insurance ownership**					
Have health insurance	2463 (73.9)	2251 (91.4)	212 (8.6)	<.001*	**2.001 (1.410-2.842)**
Not have health insurance (ref)	865 (26.1)	826 (95.5)	39 (4.5)		1
Employment status					
Employed	800 (24)	737 (92.1)	63 (7.9)	.702	1.060 (0.787-1.426)
Informal	402 (12.1)	376 (93.5)	26 (6.5)		
Formal	398 (11.9)	361 (90.7)	37 (9.3)		
Unemployed (ref)	2532 (76)	2343 (92.5)	189 (7.5)		1
Student	1362 (40.9)	1262 (92.7)	100 (7.3)		
Not employed	1170 (35.1)	1081 (92.4)	89 (7.6)		
**Needs factors**					
**Oral pain**					
Had oral pain in the previous year	1792 (53.8)	1558 (86.9)	234 (13.1)	<.001*	**12.700 (7.822-20.618)**
No oral pain in the previous year (ref)	1540 (46.2)	1522 (98.8)	18 (1.2)		1
**Caries experience**					
Had caries	1792 (53.8)	2420 (91.3)	231 (8.7)	<.001*	**3.000 (1.903-4.728)**
No caries (ref)	1540 (46.2)	660 (96.9)	21 (3.1)		1
**Psychological factors**					
Depression					
Had depression (ref)	119 (3.6)	110 (92.4)	9 (7.6)	1.000	1.000 (0.501-1.998)
No depression	3213 (96.4)	2970 (92.4)	243 (7.6)		1
Psychological distress					
Had psychological distress	142 (4.3)	127 (89.4)	15 (10.6)	.169	1.472 (0.848-2.554)
No psychological distress (ref)	3190 (95.7)	2953 (92.6)	237 (7.4)		1

Bold denotes: statistically significant.

⁎ Chi-square test.

† Binary logistic regression. Bold denotes: statistically significant.

Table 2 presents the results of the multivariable logistic regression analysis that was reported as adjusted ORs (AOR), tolerance values, and VIFs. After adjustment for all variables included in the EABM, female, urban residence, health insurance ownership, oral pain, and caries experience remained significantly associated with dental service utilization.

**Table 2:** Multivariable logistic regression between dental visits in the previous year and independent variables of youth respondents in the 2023 Indonesian Health Survey.

Variables		Dental visit in the previous year (y)		Adjusted OR (95% CI)***	Tolerance	VIF
	Total (%) (*n* = 3332)	Not visited	Visited			
**Predisposing factors**						
**Gender**						
Female	1909 (57.3)	1737 (91)	172 (9)	**1.655 (1.236-2.154)**	0.943	1.061
Male (ref)	1423 (42.7)	1343 (94.4)	80 (5.6)	1		
**Residential area**						
Urban	1927 (57.8)	1745 (90.6)	182 (9.4)	**2.274 (1.689-3.058)**	0.975	1.026
Rural (ref)	1405 (42.2)	1335 (95)	70 (5.0)	1		
Marital status						
Married	581 (17.4)	534 (91.9)	47 (8.1)	1.066 (0.743-1.528)	0.909	1.101
Divorced/single (ref)	2751 (82.6)	2546 (92.5)	205 (7.5)	1		
**Enabling factors**						
**Health insurance ownership**						
Have health insurance	2463 (73.9)	2251 (91.4)	212 (8.6)	**1.985 (1.383-2.849)**	0.992	1.008
Not have health insurance (ref)	865 (26.1)	826 (95.5)	39 (4.5)	1		
Employment status						
Employed	800 (24)	737 (92.1)	63 (7.9)	0.912 (0.663-1.256)	0.946	1.057
Informal	402 (12.1)	376 (93.5)	26 (6.5)			
Formal	398 (11.9)	361 (90.7)	37 (9.3)			
Unemployed (ref)	2532 (76)	2343 (92.5)	189 (7.5)	1		
Student	1362 (40.9)	1262 (92.7)	100 (7.3)			
Not employed	1170 (35.1)	1081 (92.4)	89 (7.6)			
**Needs factors**						
**Oral pain**						
Had oral pain in the previous year	1792 (53.8)	1558 (86.9)	234 (13.1)	**12.630 (7.747-20.582)**	0.991	1.009
No oral pain in the previous year (ref)	1540 (46.2)	1522 (98.8)	18 (1.2)	1		
**Caries experience**						
Had caries	1792 (53.8)	2420 (91.3)	231 (8.7)	**2.342 (1.467-3.741)**	0.994	1.006
No caries (ref)	1540 (46.2)	660 (96.9)	21 (3.1)	1		
**Psychological factors**						
Depression						
Had depression (ref)	119 (3.6)	110 (92.4)	9 (7.6)	1.441 (0.589-3.521)	0.637	1.569
No depression	3213 (96.4)	2970 (92.4)	243 (7.6)	1		
Psychological distress						
Had psychological distress	142 (4.3)	127 (89.4)	15 (10.6)	1.232 (0.596-2.547)	0.629	1.590
No psychological distress (ref)	3190 (95.7)	2953 (92.6)	237 (7.4)	1		

Bold denotes: statistically significant.

⁎⁎⁎ Multivariable Logistic Regression.

Female gender (AOR [95% CI] = 1.655 [1.236-2.154]; *P* < .001) and residence in urban regions (AOR [95% CI] = 2.274 [1.689-3.058]; *P* < .001) were associated with an increased likelihood of visiting a dental health practitioner compared to their counterparts. Marital status had not a significant association with the utilization of dental services. Health insurance holders (AOR [95% CI] = 1.985 [1.383-2.849]; *P* < .001) were significantly more likely to utilize dental services compared to those without insurance. Conversely, employment status has shown insignificant association with dental services. In this study, the highest probability associated with dental service utilization was shown by need factors, namely oral pain (AOR [95% CI] = 12.630 [7.747-20.582]; *P* < .001) and caries experience (AOR [95% CI] = 2.342 [1.467-3.741]; *P* < .001). This may indicate that adolescents and young adults who experience oral symptoms are much more likely to use dental services. After adjustment, for both psychosocial factors, depression and psychological distress showed insignificant associations with dental service utilization. Respondents with depression had 1.44 times higher odds of utilizing dental services than those without depression (AOR [95% CI] = 1.441 [0.589-3.521]), while psychological distress was also not significantly associated with dental visits (AOR [95% CI] = 1.232 [0.596-2.547]).

In contrast, marital status, employment status, and emotional mental disorder were not significantly associated with dental service utilization after adjustment.

Multicollinearity diagnostics indicated no evidence of substantial collinearity among the independent variables. Tolerance values ranged from 0.629 to 0.992, whereas VIF values ranged from 1.006 to 1.590, all of which were within commonly accepted thresholds.

### Factor analysis

Exploratory factor analysis was performed to ascertain the underlying structure of factors related to dental service utilization. *The* KMO measure of sampling adequacy was 0.516, signifying adequate sufficiency for factor analysis, while Bartlett’s test of sphericity was statistically significant (*χ*² = 2978.925; df = 36; *P* < .001), affirming the correlation matrix’s suitability for factor extraction.

PCA utilizing varimax rotation revealed four components with eigenvalues exceeding 1.0, which collectively explained 60.24% of the total variance. The initial component, representing the psychosocial domain, comprised 19.50% of the variation and was mostly influenced by characteristics related to depression and psychological distress. The second component represented 15.88% of the variation and was significantly associated with oral pain/dental complaints and caries experience, signifying need-related factors. The third component represented 13.14% of the variance and was predominantly comprised of gender and marital status variables, which aligned with predisposing factors. The fourth component represented 11.72% of the variance and was mostly influenced by health insurance ownership and residential area, which are enabling factors. The rotated component matrix indicated substantial factor loadings for depression (0.916) and psychological distress (0.915) in Component 1; oral pain/dental complaints (0.800) and caries experience (0.644) in Component 2; gender (0.778) and marital status (0.744) in Component 3; and health insurance ownership (0.762) and residential area (−0.682) in Component 4.

Given the marginal KMO values and the categorical nature of the variables, these findings are intended to provide initial insights into the clustering of variables rather than to validate the model structure. Thus, the factor structure should be interpreted with caution. The PCA results provide exploratory evidence that the variables cluster in a manner consistent with the conceptual areas of EABM, but they do not validate the model structure (Table 3, Table 4).

**Table 3:** Principal component analysis of factors associated with dental service utilization among Indonesian youth.

Variables	Communality	Component 1 (psychosocial)	Component 2 (need factors)	Component 3 (predisposing factors)	Component 4 (enabling factors)
Depression	0.842	**0.916**	0.032	0.041	−0.018
Psychological distress	0.839	**0.915**	0.025	0.038	−0.012
Oral pain/dental complaints	0.687	0.071	**0.800**	0.046	0.021
Caries experience	0.521	0.052	**0.644**	0.083	−0.041
Gender	0.615	0.033	0.021	**0.778**	0.072
Marital status	0.573	0.045	0.014	**0.744**	−0.038
Health insurance ownership	0.601	−0.026	0.058	0.043	**0.762**
Residential area	0.497	0.038	−0.041	0.071	**−0.682**

PCA with varimax rotation was performed. Factor loadings ≥0.40 are shown in bold. Four components with eigenvalues >1.0 were retained. The four-component solution explained 60.24% of the total variance. Communality values represent the proportion of variance in each variable explained by the extracted components.

**Table 4:** Integrative interpretation of findings based on EABM.

Andersen model domain	Variables	Statistical findings	Factor analysis findings	Integrated interpretation
**Predisposing factors**	Gender	Female respondents were more likely to utilize dental services than males (AOR = 1.655; 95% CI: 1.236-2.154; *P* < .001).	Gender showed a strong loading in Component 3 (0.778).	Predisposing characteristics associated with utilization behaviour, particularly gender-related differences in healthcare-seeking behaviour. Female respondents may demonstrate greater oral health awareness and preventive orientation than males.
	Residential area	Urban respondents were more likely to utilize services than rural respondents (AOR = 2.274; 95% CI: 1.689-3.058; *P* < .001).	Residential area loaded strongly in Component 4 (−0.682).	Geographic disparities remain an important factors of utilization. Urban residence likely reflects better physical access, availability of providers, and fewer structural barriers.
	Marital status	No significant association with utilization (*P* = .597).	Marital status demonstrated a substantial loading in Component 3 (0.744).	Although marital status conceptually associated with the predisposing domain, it did not exert a measurable independent association on utilization behaviour among youth respondents.
**Enabling factors**	Health insurance ownership	Respondents with insurance were more likely to utilize services (AOR = 1.985; 95% CI: 1.383-2.849; *P* < .001).	Health insurance ownership loaded strongly in Component 4 (0.762).	Enabling factors played an important role in facilitating access to dental care. Insurance coverage may reduce financial barriers; however, utilization remained relatively low overall, indicating that coverage alone is insufficient to ensure preventive service use.
	Employment status	No significant association with utilization (AOR = 0.912; 95% CI:0.663-1.256; *P* = .702).	Employment status was not retained as a dominant component.	Employment status may have limited relevance among youth populations, particularly because many respondents were students or economically dependent.
**Need factors**	Oral pain	Strongest association with utilization (AOR = 12.630; 95% CI: 7.747-20.582; *P* < .001).	Dental complaints loaded strongly in Component 2 (0.800).	Need factors emerged as the strongest factors of utilization, indicating that dental attendance was predominantly symptom-driven rather than preventive.
	Caries experience	Significant association with utilization (AOR = 2.342; 95% CI: 1.467-3.741; *P* < .001).	Caries experience contributed to Component 2 (−0.553).	Existing oral disease increased the likelihood of seeking care, reinforcing the predominance of curative-oriented utilization patterns.
**Psychosocial factors**	Depression	No significant association with utilization (AOR 1.441; 95% CI 0.589-3.521; *P* = 1.000).	Depression demonstrated a very strong loading in Component 1 (0.916).	Although depression was in line with psychosocial construct, it did not directly related to dental service utilization. This suggests that psychosocial vulnerability may exist without translating into observable healthcare-seeking behaviour.
	Psychological distress	No significant association with utilization (AOR 1.232; 95% CI 0.596-2.547; *P* = .169).	Psychological distress also loaded strongly in Component 1 (0.915).	Psychosocial factors were conceptually related but appeared less associative than structural and clinical factors in shaping utilization behaviour within this context. Possible explanations include underreporting, stigma, and the predominance of symptom-driven care-seeking.

### Overall integrative interpretation

The logistic regression and factor analysis findings were broadly consistent with the conceptual domains of the EABM. The results suggest that variables representing enabling and need-related domains were more strongly associated with dental service utilization than those representing psychosocial domains. While psychosocial variables clustered together statistically, they were not significantly associated with dental service utilization in the adjusted model. These findings may indicate that, within the Indonesian context, dental service utilization among youth is more closely related to structural access factors and perceived oral health needs than to the psychosocial factors measured in the 2023 IHS. Nevertheless, the absence of significant associations should be interpreted cautiously, as it might reflect limitations in measurement sensitivity, underreporting, or indirect pathways through which psychosocial factors influence oral health behaviours and care-seeking practices.

## Discussion

Dental service utilization among Indonesian youth was notably low, with only 7.6% of respondents reporting a dental visit in the previous year. This rate was significantly lower than in other developing countries, including South Africa and Iran.^17^^,^^18^ In South Africa, 30% of those aged 15 to 19 had seen a dental health practitioner in the previous year.^17^ In Iran, 50.7% of the 15 to 19 age group had visited a dental health practitioner in the previous year.^18^ Although there is an increase compared to a national health survey in 2018 which estimated that only 4% of Indonesians visit a dentist within the past year,^19^ this finding indicates persistent barriers to oral healthcare access and suggests that preventive dental attendance remains limited among young populations in Indonesia. The low utilization rate of oral health services in Indonesia (as a low-middle income country) might be related to limited access to oral health services, an inadequate oral health care system, or individual characteristics.^20^ Based on the SKI 2023 report, 81.4% of the total population of Indonesia knew of the existence of clinics or independent practices run by health practitioners, but 63.6% found these clinics and practices difficult to be accessed^12^ although the density of dentistry personnel (per 10 000 population) has improved from 0.2 in 2003 to 1.2 in 2024.^21^

When interpreted using EABM, the present study demonstrated that dental service utilization was primarily associated with enabling and need factors, while psychosocial factors showed limited association on utilization behaviour. These findings underscore the importance of contextualizing behavioural models within specific healthcare environments, particularly in low- and middle-income countries undergoing healthcare transformation. Similar applications of the Andersen model in Mexico,^3^ India^10^ and Brazil^11^ also reported that structural access and clinical need were closely related factors to dental service utilization among adolescents and young adults.

### Predisposing factors

Among the predisposing factors, Gender had a significant association (*P* < .001) with dental service utilization: female respondents were more likely to utilize dental health services than males. This finding is consistent with previous studies among adolescents in Mexico,^3^ Saudi Arabia^22^ and USA,^23^ which reporting that female adolescents were more likely to access dental services than males. Female group generally demonstrate greater health awareness, stronger preventive orientation, and more positive healthcare-seeking behaviour than men.^3^^,^^22^^,^^23^ These studies also found that female adolescents and young adults may also be more attentive to oral appearance and oral health-related quality of life, which could increase the likelihood of seeking professional dental care.

Residential area was also significantly associated with utilization, with urban respondents demonstrating a higher likelihood of visiting dental health services compared with rural respondents. This disparity likely reflects unequal distribution of oral healthcare facilities and workforce availability between urban and rural areas in Indonesia. Similar findings have been reported in Saudi Arabia^22^ as high-income country, and other middle-income settings such as India^10^ and Brazil^11^ as well as Bosnia-Herzegovina as a European developing country,^24^ where contextual and geographic inequalities substantially influence oral healthcare utilization. Urban populations may benefit from shorter travel distances, greater service availability, and improved health information exposure, whereas rural populations often face structural barriers including transportation limitations and provider shortages. Furthermore, in Indonesian context, there were financial limitations and a lack of oral health facilities in rural areas, including disparities in the distribution of dental health practitioners.^25^

Although marital status contributed conceptually to the predisposing domain in the factor analysis, it was not significantly associated with utilization behaviour. This may reflect the relatively homogeneous age group included in the study, as most respondents were unmarried adolescents or young adults, thereby limiting variability in marital-related behavioural patterns. However, marital status should still be a factor to consider because it is closely related to the utilization of dental health services, as stated in a systematic review that spouses can provide support and motivation to improve oral health; hence, the level of oral health service utilization in individuals who are divorced or single is lower than that in individuals who are married.^26^

### Enabling factors

Health insurance ownership emerged as an important enabling factor associated with dental service utilization. Respondents with health insurance were approximately twice as likely to utilize dental services compared with uninsured respondents. This finding supports previous evidence indicating that financial protection mechanisms somehow facilitate access to oral healthcare services.^27^ A systematic review of studies using the Andersen model reported that enabling resources, particularly financial access and insurance coverage, consistently influenced dental service utilization.^9^ Health insurance can alleviate the financial burden, which is the primary obstacle preventing persons from seeking dental care, particularly in impoverished and lower-middle-income populations.^28^ Within the Indonesian context, the implementation of the National Health Insurance system (JKN) may have contributed to improved accessibility of dental services among insured populations. Nevertheless, despite the positive association between insurance ownership and utilization, the overall utilization rate remained low. This indicates that insurance coverage alone may be insufficient to promote preventive dental attendance, especially public health insurance in Indonesia.^27^ Other barriers, including limited awareness of covered dental benefits, low perceived need for preventive care, and restricted provider availability, may continue to limit utilization. Similar patterns have been reported in studies from Brazil^11^ and Mexico,^3^ where enabling resources improved access but did not fully eliminate disparities in utilization.

Employment status was not significantly associated with utilization behaviour. This finding may reflect the characteristics of the study population, as many respondents within the 15- to 24-year age group were likely students or financially dependent on their families, thereby reducing the direct influence of employment-related economic factors on healthcare utilization. Nevertheless, employment status still should be our consideration since the 2023 IHS report stated that 76.7% of Indonesians did not visit dental health professionals due to lack of funds.^29^ This hesitation might be triggered by the assumption that a visit to the dentist requires a large amount of money, so that unemployed people generally choose not to visit the dentist.

### Need factors

Need factors demonstrated the strongest associations with dental service utilization in this study. Respondents who experienced oral pain were substantially more likely to seek dental care, while caries experience was also significantly associated with utilization. A previous study among student in Indonesia also revealed that toothache experience had significant association with dental visit.^30^ These findings suggest that dental attendance among Indonesian youth remains predominantly symptom-driven rather than preventive. The strong association between oral pain and service utilization may indicate that many individuals seek treatment only after symptoms become severe enough to interfere with daily activities. Most people typically visit a dental health practitioner only when they experience a problem with their teeth and/or mouth; otherwise, they will not schedule an appointment with the practitioner.^31^ Similarly, respondents with caries experience were more likely to utilize services because treatment is often initiated after visible disease progression or discomfort occurs. This reactive pattern of care-seeking behaviour reflects missed opportunities for early prevention and routine dental examinations. These findings are consistent with studies conducted in India,^32^ Brazil,^11^ and Mexico,^3^ where oral healthcare utilization among adolescents was frequently motivated by urgent treatment needs rather than preventive awareness. A systematic review of dental service utilization based on the Andersen model also concluded that need factors, including oral health problems and perceived disease severity, were among the strongest predictors of utilization behaviour.^9^ Without a shift towards preventive-oriented utilization, oral healthcare systems may continue to face disproportionate burdens related to advanced disease management and curative treatment costs.

### Psychosocial factors

Notably, depression and psychological distress were not significantly associated with dental service utilization in the regression analysis. Depression had no significant association with having made a dental visit in the previous year. Anttila et al^33^ had similar findings, but Choi et al presented contrasting findings. Choi et al^34^ stated that individuals who have depression were 2.262 times more likely to never visit a dental health practitioner than those without depression. For depression, the adjusted OR was higher than the crude estimate, suggesting that the relationship between depression and dental service utilization may have been partially masked by other demographic, enabling, or need-related factors. Nevertheless, the association remained statistically nonsignificant, indicating insufficient evidence for an independent relationship in the present study. Regarding psychological distress, a similar finding from study by Gaffar et al^35^ stated that dental anxiety had no significant association with having made a dental visit in the previous year. Few previous study directly address the association between dental visits and psychological distress. Nermo et al^36^ stated that psychological distress is related to dental anxiety, and it is well-known that dental anxiety has an important influence on dental visits, while Su et al^37^ concluded that psychological distress partly explains the association of financial strain with dental attendance. However, factor analysis showed that the two variables appeared consistent in forming distinct psychosocial constructs with very strong factor loadings. This finding suggests that the psychosocial variables seem to be conceptually coherent and in line with the psychosocial domain, despite the lack of a direct relationship between them and utilization behaviour. Psychosocial factors may also indirectly affect service utilization via perceived oral health or self-care habits, rather than through direct service utilization. This necessitates additional research in the future.

The apparent discrepancy between regression findings and factor analysis does not necessarily indicate contradictory results, as both analyses address different analytical objectives. Logistic regression evaluates whether psychosocial variables directly predict utilization behaviour, whereas factor analysis assesses whether these variables cluster together conceptually as part of a latent psychosocial construct. Therefore, the findings indicate that psychosocial vulnerability may exist among respondents without necessarily translating into observable healthcare-seeking behaviour. Several explanations may account for this pattern. In the Indonesian context, utilization of dental services among youth may be more strongly driven by immediate clinical needs and structural access factors than by psychological conditions. Oral pain and caries experience demonstrated substantially stronger associations with utilization, suggesting that treatment-seeking behaviour remains largely reactive and symptom-oriented. In addition, mental health conditions among adolescents and young adults may be underrecognized or underreported because of social stigma, limited mental health literacy, and cultural barriers surrounding psychological disclosure. These findings further highlight the importance of distinguishing between conceptual validity of psychosocial constructs and their observable behavioural effects within EABM.

The combined findings from logistic regression and factor analysis suggest that EABM might be conceptually supported in this study, as the variables clustered into coherent theoretical domains. Nevertheless, the relative contribution of each domain differed statistically. Need factors and enabling factors exerted the strongest observable association on dental service utilization, whereas psychosocial factors demonstrated a weaker behavioural association despite forming a coherent construct.

Overall, these findings suggest that dental service utilization among Indonesian youth is more associated with structural access and immediate clinical needs than by psychosocial conditions. This pattern may reflects a predominantly curative-oriented utilization model, in which healthcare services are sought primarily in response to symptoms rather than as part of routine preventive care. Hence, we suggest a number of practical programs, including expanding access to preventive oral healthcare in rural and underserved areas, raising awareness about dental benefits through the national insurance system (JKN), and incorporating oral health promotion into larger youth health initiatives.

Given the low utilization of dental health services among Indonesian youth, future oral health promotion initiatives should be tailored specifically to the 15 to 24 age group. Digital health literacy interventions delivered through social media platforms, mobile apps, and online educational campaigns may be particularly effective, given the high levels of digital engagement among adolescents and young adults. For instance, a previous study by Novrinda et al^38^ found that online video educational intervention seems to have great effect in increasing knowledge among university students in Indonesia. Furthermore, school- and university-based oral health programs may be able to facilitate routine checkups, preventative education, and referral pathways to professional dental services. Such approaches may complement existing JKN initiatives and help promote preventative rather than symptom-driven dental health service utilization patterns.

The overall findings of this study should be viewed in light of its limitations. This study is the use of secondary data, which only allows for processing of readily available data. Some socioeconomic variables potentially linked with dental service utilization, such as household income and parental educational background, were not included in this study; thus, residual confounding is still conceivable.

Subjectivity in the responses of participants is likely given that several variables were self-administered questionnaire. Another limitation is the cross-sectional design, which does not allow for establishing causal relationships. The PCA results should be interpreted cautiously because several included variables were binary in nature and the KMO value indicated only marginal sampling adequacy. Consequently, the analysis was intended to provide exploratory insights into latent variable clustering rather than formal construct validation.

Nevertheless, this study includes nationally representative data. Therefore, its findings may serve as a reference for national health policy, particularly among youth. In addition, the caries experience was assessed through clinical oral examination conducted within the 2023 IHS rather than relying solely on self-reported information. This enhances the validity of the need-factor assessment and reduces the risk of reporting bias commonly observed in population-based oral health research. Another strength was the presentation of EABM through an applicative and also a conceptual approach, may provide new insights for utilization research in the future. Future longitudinal studies are warranted to examine whether psychosocial vulnerability influences dental service utilization over time. Such designs may better capture temporal relationships between depression, psychological distress, oral health perceptions, self-care behaviours, and subsequent healthcare-seeking patterns that cannot be adequately assessed using cross-sectional data.

## Conclusions

Dental service utilization based on the expanded Andersen behavioural model among Indonesian youth remains low and was associated significantly with gender and residential area (**predisposing**), health insurance ownership (**enabling**), oral pain and caries experience (**needs**), while **psychosocial** domain had insignificant association. Dental service utilization was primarily driven by clinical need and structural access factors rather than psychosocial conditions. While psychosocial variables might form a coherent latent construct, they did not translate into observable utilization behaviour. These findings highlight a predominantly symptom-driven pattern of care and underscore the need for policies that strengthen preventive oral health promotion, improve awareness of insurance benefits, and address geographic disparities in access to dental care.

## Funding

This work was supported by the Directorate of Research and Development, Universitas Indonesia under Hibah PUTI Q1 2024 (Grant no. NKB 330/UN2.RST/HKP.05.00/2024).

## Declaration of generative AI and AI-assisted technologies in the writing process

During the preparation of this work, the authors used TRINKA AI (as provided officially by Universitas Indonesia) in order to paraphrase several sentences. After using this tool/service, the authors reviewed and edited the content as needed and take full responsibility for the content of the publication.

## Author contributions

*Conceptualized the study, collected and analyse the data, participated in manuscript writing, revising it critically for important intellectual content, and funding acquisition:* Novrinda*. Conceptualized the study, collected and analyse the data, and participated in manuscript writing:* Irsan*. Conceptualized the study, participated in manuscript writing, revising it critically for important intellectual content, and funding acquisition:* Ramadhani*.* Participated in manuscript writing and revising it critically for important intellectual content, and funding acquisition: Darwita, Dong-Hun*.* Participated in manuscript writing and revising it critically for important intellectual content: Badruddin, Bahar. *Reviewed and approved the final version of the manuscript:* All authors.

## Conflict of interest

The authors declare that they have no known competing financial interests or personal relationships that could have appeared to influence the work reported in this article.
